# Polysaccharides of* Fructus corni* Improve Ovarian Function in Mice with Aging-Associated Perimenopause Symptoms

**DOI:** 10.1155/2019/2089586

**Published:** 2019-06-27

**Authors:** Yong Wang, Jing-zhen Wu, Yu Li, Xu Qi

**Affiliations:** ^1^Department of Pharmacy, Jiangsu Jianhu People's Hospital, Yancheng 224700, China; ^2^School of Medicine and Life Sciences, Nanjing University of Chinese Medicine, Nanjing 210023, China; ^3^Department of Respiratory Medicine, The First Affiliated Hospital of Nanjing Medical University, Nanjing 210029, China

## Abstract

**Objective:**

Perimenopause symptoms have an extremely high incidence in aging women. Development of new strategies to improve perimenopause symptoms is important topic in clinical context. Increasing studies have shown that the polysaccharides of* Fructus corni* (PFC) have many pharmacological activities including antiaging effects. Here, we evaluated the effects of PFC on the ovarian function in natural aging-associated perimenopause symptoms in mice.

**Methods:**

Natural aging mice (16-month old) were orally administrated with PFC at 1.11 g/kg daily for 24 days with none-treated young mice (3-month old) as control. Blood samples were collected for measurements of serum levels of estradiol, progesterone, luteinizing hormone (LH), and follicle stimulating hormone (FSH). Ovaries were isolated for histopathological and molecular exanimations.

**Results:**

We found that the aging mice had decreased number of growing follicles and corpus luteum in ovary, but treatment with PFC restored their amounts. Measurement of hormones showed that there were low serum levels of estradiol and progesterone but high levels of LH and FSH in aging mice; however PFC restored estradiol and progesterone levels but reduced LH and FSH levels. Immunohistochemical analysis with ovarian tissues also revealed that the expression of inhibin and insulin-like growth factor 1 was reduced in the ovary of aging mice but was restored by PFC. These data indicated that PFC regulated ovarian function-associated hormone levels in aging mice. Furthermore, there was reduced expression of antiapoptotic protein Bcl-2 and increased expression of proapoptotic molecules Bax and cleaved-caspase-3 in the ovary of aging mice. However, treatment with PFC upregulated Bcl-2 and downregulated Bax and cleaved-caspase-3, suggesting that PFC inhibited apoptosis of granulosa cells in the ovary of aging mice.

**Conclusion:**

PFC improved the ovarian function in mice, which had high potential to be developed as a safe and effective therapeutic remedy for aging-associated perimenopause symptoms.

## 1. Introduction

Perimenopause, also known as the menopausal transition, defines a period of time during which a series of physiological alterations mark progression toward the final menstrual period of a woman. This transition starts with the onset of menstrual irregularities and continues until menopause has occurred, which may last for a variable amount of time with a median of four years [1]. During perimenopause, a woman may suffer from a number of symptoms, including menstrual cycle changes, insomnia, dysphoric mood symptoms, and somatic symptoms [2]. It is estimated that as many as 90% of women will ask for advice on how to control or relieve these menopausal-associated symptoms, suggesting that perimenopause symptoms are important topics in the clinical practice worldwide [3].

Clear evidence has demonstrated that perimenopausal women commonly suffer from ovarian dysfunction, resulting in systemic changes of hormones mainly including estradiol and progesterone [4]. A number of basic and clinical studies have evaluated the use of hormone replacement therapy (HRT) for perimenopause symptoms [5]. Despite the therapeutic benefits of HRT, risks or problems also occur in some subpopulation in women [6]. On the other hand, understanding the pathophysiology of ovarian function decline can help guide clinical management. Granulosa cells (GCs) play an important role in the growth and development of the follicle in the process known as folliculogenesis. The major functions of GCs include the production of sex steroids, as well as myriad growth factors thought to interact with the oocyte during its development [7]. Emerging evidence suggests that apoptosis of GCs is concomitant with aging-associated ovarian function decline leading to ovarian hormone secretion disorder [8]. Therefore, prevention of GCs apoptosis represents a novel strategy for treatment of perimenopause symptoms.

In recent years, there has been renewed interest in the potential of purified natural products to provide health and medical benefits and to prevent disease.* Fructus corni* is one of the most common traditional Chinese herbal medicines used as a common option for liver and kidney nourishing, in which polysaccharides are characterized to be the main functional components and have attracted accumulated attentions [9]. Pharmacological studies have demonstrated that the polysaccharides of* Fructus corni *(PFC) have a wide range of bioactivities, such as immunomodulatory, antioxidant, antitumor, and antiaging effects [10]. Herein, we aimed to evaluate the effects of PFC on ovarian functions in naturally aging female mice, in the hoping of revealing the therapeutic potential of PFC for women experiencing perimenopause symptoms.

## 2. Methods

### 2.1. Reagents and Antibodies

PFC (purity 95%) was purchased from Ningbo Dekang Biological Products Co., Ltd. (Ningbo, China). The primary antibodies against Bcl-2, Bax, cleaved-caspase-3, and *β*-actin for Western blot assays were obtained from Cell Signaling Technology (Danvers, MA, USA). The primary antibodies against inhibin, IGF-1, Bcl-2, Bax, and cleaved-caspase-3 for immunohistochemistry were provided by Proteintech Group (Chicago, IL, USA).

### 2.2. Animal Experiments

All animal experiments were approved by the Institutional Animal Care and Use Committee [protocol number ACU-20(20151201)], Nanjing University of Chinese Medicine, and conformed to the Guide for the Care and Use of Experimental Animals. Ten young female ICR mice (3-month old, body weight 20±2 g ) and 20 aging female ICR mice (16-month old, body weight 50±5 g) were provided by the Experimental Animal Center of Nanjing University of Chinese Medicine. All mice were housed in plastic cages (5 mice per cage) and maintained in standardized conditions at 20 ± 2°C room temperature, 40 ± 5% relative humidity, and a 12 h light/dark cycle. Water and pelleted food were available ad libitum. Animals were acclimated to the animal facility for 1 week. The 20 aging mice were randomly divided into two groups: aging model group (n=10) and PFC intervention group (n=10). The 10 young mice were set as control group. The mice in PFC intervention group were administrated with PFC at a dose of 1.11 g/kg daily by gavage for continuous 24 days. This dose used in mice was calculated from human clinical doses based on the body weight and body surface area in accordance with the published methods [11]. The young control mice and aging model mice were given normal saline daily by gavage (0.5 ml/mouse) for continuous 24 days. Of note, the estrous phase of each mouse was checked by vaginal smears before grouping and during the experiments according to reported methods [12]. The mice with regular estrous phase were used for experiments, and we made sure that the mice in each group at similar estrus stages when they were euthanized. At the end of experiments, all mice were anaesthetized by inhalation of ether followed by blood collection via retro orbital sinus. Then, mice were killed by decapitation followed by isolation of bilateral ovaries for histopathological and molecular exanimations.

### 2.3. Hematoxylin-Eosin (HE) Staining

Mouse left ovaries were fixed in 10% neutral buffered formalin. After washing with phosphate-buffered saline, the fixed tissues were dehydrated in graded ethanol and embedded in paraffin. Five serial paraffin sections (5 *μ*m thickness of each) were sliced from each sample using a sliding microtome, and paraffin was removed using xylene. Tissue sections were stained with HE reagents according to standard procedures [13]. Images were blindly taken at random fields under a microscope (Nikon, Tokyo, Japan). The number of growing follicles and corpus luteum per field was counted. Representative views are shown.

### 2.4. Measurements of Serum Hormone Levels

Blood samples from mice were incubated at room temperature for 1 h to allow clotting. Serum was extracted after centrifugation and aliquoted. Serum levels of estradiol, progesterone, luteinizing hormone (LH), and follicle stimulating hormone (FSH) were measured using their corresponding radioimmunoassay kits (Tianjin Jiuding Medical Bioengineering Co., Ltd., Tianjin, China) according to the manufacturer's instructions.

### 2.5. Real-Time PCR

Total RNA was isolated from mouse right ovarian tissues using TRIzol reagent (Invitrogen, Carlsbad, CA, USA). First-strand cDNA was synthesized with 1 *μ*g of total RNA using a PrimeScript RT reagent kit (TakaraBio, Tokyo, Japan). The quantitative real-time PCR was performed using IQTM SYBR Green supermix and the iQ5 real-time detection system (Bio-Rad Laboratories, Hercules, CA, USA). Reaction mixtures contained 7.5 *μ*l of SYBR Green I dye master mix (Quanta), 2 pM each of forward and reverse primers. Thermocycler conditions included initial denaturation at 50°C and 95°C (10 min each), followed by 40 cycles at 95°C (15 s) and 60°C (1 min). Glyceraldehyde phosphate dehydrogenase (GAPDH) was used as the invariant control mRNA abundance was determined by 2^−ΔΔCT^ method [14]. The primers of genes (GenScript, Nanjing, China) were as follows: Bcl-2: (forward) 5′-CCCACCTGTGGTCCATCTGAC-3′, (reverse) 5′-CGGTAGCGACGAGAGAAGTC-3′; Bax: (forward) 5′-CTGGATCCAAGACCAGGGTG-3′, (reverse) 5′-GGGGTCCCGAAGTAGGAGAG-3′; GAPDH: (forward) 5′-TGACAACAGCCTCAAGAT-3′, (reverse) 5′-GAGTCCTTCCACGATACC-3′. Results were from triplicate experiments.

### 2.6. Western Blot Analyses

Protein abundance was detected using Western blot analyses according to the reported procedures [15]. Briefly, total lysates from mouse right ovarian tissues were prepared with RIPA buffer (50 mM Tris, pH 7.2; 150 mM NaCl; 0.5% sodium deoxycholate; 0.1% sodium dodecyl sulfate; 1% Nonidet P-40; 10 mM NaF; 1 mM Na_3_VO_4_; protease inhibitor cocktail). Lysates were sonicated for 10 s and centrifuged at 14, 000 rpm for 10 min at 4°C. Protein concentrations were determined by bicinchoninic acid assay with BSA as a standard (Pierce, Rockford, IL, USA). Equivalent amounts of protein (50 *μ*g/lane) were separated on 7.5%-12% SDS-polyacrylamide gel and transferred to polyvinylidene difluoride membranes (Millipore, Bedford, MA, USA). Membranes were incubated with phosphate buffer solution containing 0.05%Tween 20 and 5% nonfat dry milk to block nonspecific binding and were incubated with primary antibodies (dilution 1:1000), then with appropriate secondary antibodies conjugated to horseradish peroxidase (dilution 1:10000). Immunoreactive bands were visualized by using Renaissance chemiluminescence reagent (Perkin-Elmer Life Science, Boston, MA, USA). *β*-actin was used as an invariant control for equal loading of total proteins. The levels of target protein bands were densitometrically determined using Image Lab Software 3.0. The variation in the density of bands was expressed as fold changes compared to the control in the blot after normalization to *β*-actin. Presented blots are representative of three independent experiments.

### 2.7. Immunohistochemistry

Five serial sections (5 *μ*m thickness of each) of mouse left ovarian tissues were incubated with the primary antibodies against inhibin (dilution 1: 100), IGF-1 (dilution 1: 100), Bcl-2 (dilution 1: 100), Bax (dilution 1:100), or cleaved-caspase-3 (dilution 1:100) for immunohistochemical analysis using standard methods [16]. Images were blindly taken at random fields under a microscope (Nikon, Tokyo, Japan). For each slice, five fields were randomly selected for quantitative analysis. Image Pro Plus 6.0 software (Media Cybernetics, Rockville, MD, USA) was used to calculate the positive staining area (%). Representative views are shown.

### 2.8. Statistical Analysis

Data were presented as mean ± SD, and results were analyzed using SPSS16.0 software. The significant differences of normally distributed data was determined by one-way ANOVA with post hoc Tukey's test for comparison between multiple groups under the condition that F achieved* P* < 0.05, and there was no significant variance inhomogeneity. For the nonnormally distributed data, Kruskal-Wallis H test was used to determine significant differences between multiple groups. Values of* P* < 0.05 were considered to be statistically significant.

## 3. Results

### 3.1. PFC Improves Ovarian Histology and Increases Follicles and Corpus Luteum in Aging Mice

**Figure 1:**
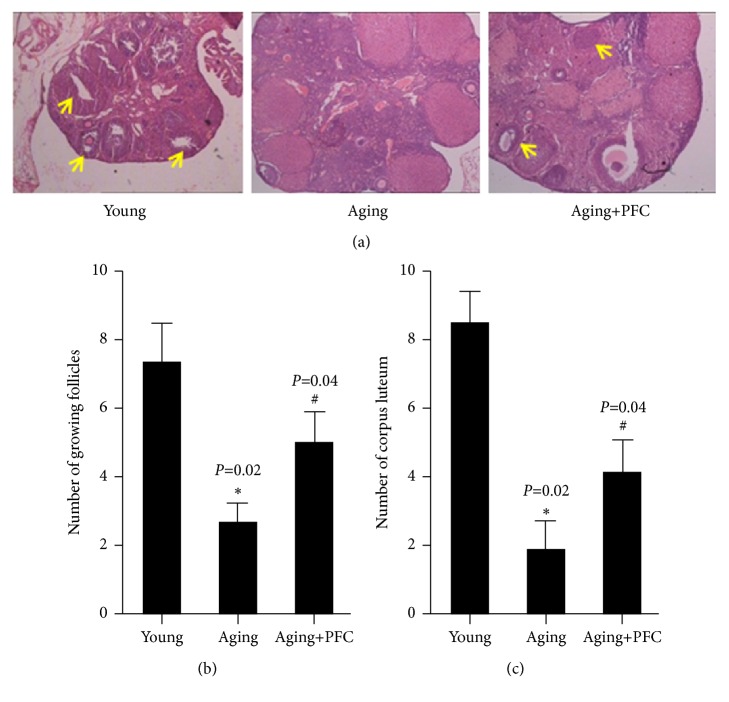
PFC improves ovarian histology and increases follicles and corpus luteum in aging mice. (a) HE staining with ovarian tissues. The yellow arrows indicate the growing follicles. (b) Quantification of growing follicles in ovarian tissues. (c) Quantification of corpus luteum in ovarian tissues. For statistical significance in this figure, *∗P* < 0.05 compared with the young control group; ^#^*P* < 0.05 compared with the aging model group; Kruskal-Wallis H test.

We initially examined the histology of mouse ovary. Compared with the young control mice, there were less ovarian follicles and corpus luteum in ovarian cortex, but more atresia follicles in aging mice; however, treatment with PFC recovered these histological features to a certain extent compared with the aging model mice (Figure 1(a)). Consistently, quantification of growing follicles showed that the number of growing follicles was significantly decreased in the ovary of aging model mice, which was remarkably recused by treatment with PFC (Figure 1(b)). Moreover, the number of corpus luteum was significantly decreased in the ovary of aging model mice, but administration of PFC significantly restored the amount of corpus luteum (Figure 1(c)). Altogether, these data indicated that PFC improved ovarian histology and restored follicles and corpus luteum in aging mice.

### 3.2. PFC Regulates Ovarian Function-Associated Hormone Levels in Aging Mice

**Figure 2:**
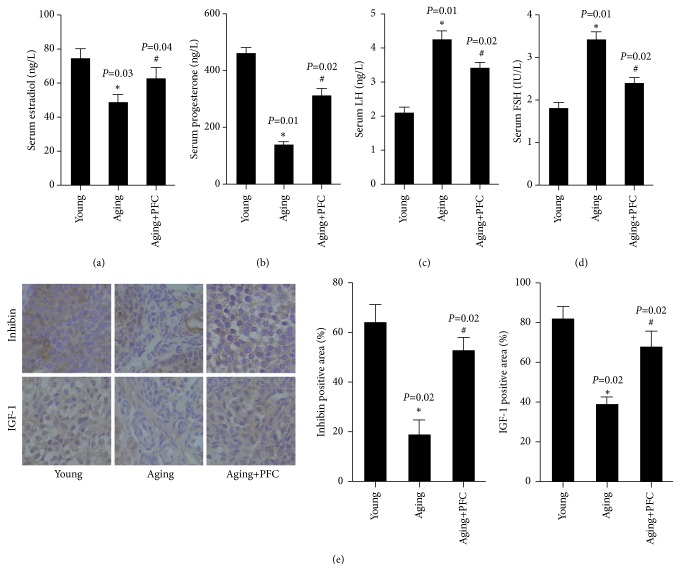
PFC regulates ovarian function-associated hormone levels in aging mice. (a) Serum estradiol levels. (b) Serum progesterone levels. (c) Serum LH levels. (d) Serum FSH levels. (e) Immunohistochemical analyses of inhibin and IGF-1 in mouse ovarian tissues with quantification of positive staining area. For statistical significance in this figure, *∗P* < 0.05 compared with young control group; ^#^*P* < 0.05 compared with aging model group; one-way ANOVA with post hoc Tukey's test for (a-d); Kruskal-Wallis H test for (e).

We next determined the alterations in the hormone levels associated with ovarian function. We observed that the serum levels of estradiol and progesterone were significantly declined in aging model mice compared to the young control mice, but their serum concentrations were significantly rescued by PFC (Figures 2(a) and 2(b)). In addition, the serum levels of LH and FSH were significantly increased in aging model mice compared to the young control mice, but PFC intervention effectively reduced their serum concentrations in aging mice (Figures 2(c) and 2(d)). Immunohistochemical analysis with ovarian tissues additionally showed that the ovarian expression of inhibin and insulin-like growth factor 1 (IGF-1) were considerably downregulated in aging model mice but were restored by treatment PFC (Figure 2(e)). Taken together, PFC regulated ovarian function-associated hormone levels in aging mice.

### 3.3. PFC Prevents Apoptosis of Ovarian GCs in Aging Mice

**Figure 3:**
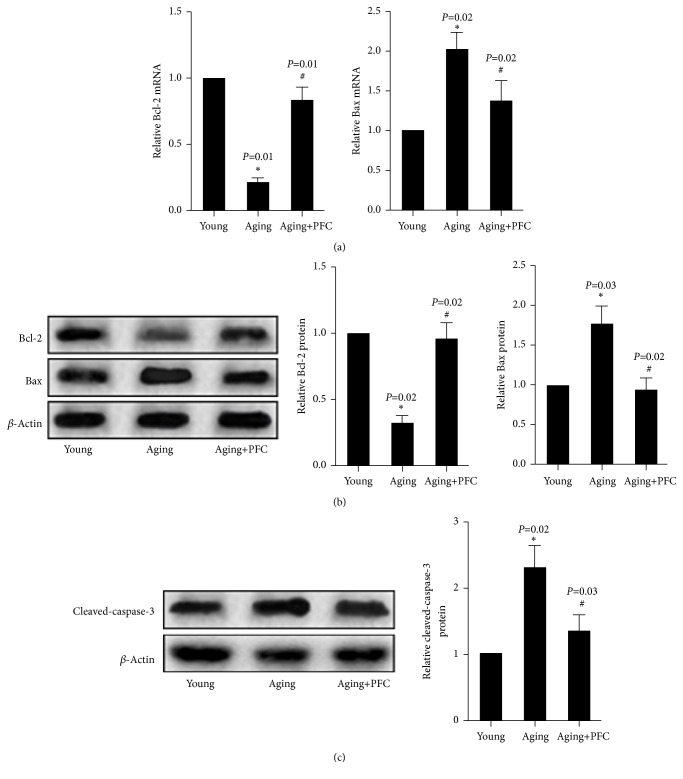
PFC prevents apoptosis of ovarian GCs in aging mice. (a) Real-time PCR analyses of mRNA expression of Bcl-2 and Bax in ovarian tissues. (b) Western blot analyses of protein abundance of Bcl-2 and Bax in ovarian tissues with quantification. (c) Western blot analyses of protein abundance of cleaved-caspase-3 in ovarian tissues with quantification. For statistical significance in this figure, *∗P* < 0.05 compared with young control group; ^#^*P* < 0.05 compared with aging model group; Kruskal-Wallis H test.

**Figure 4:**
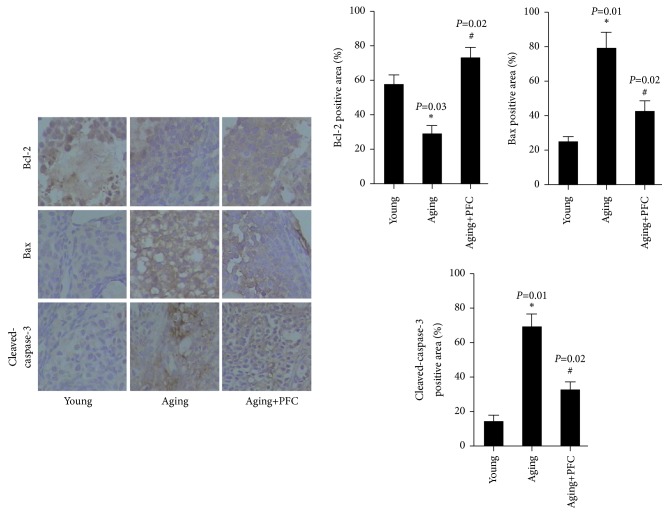
Immunohistochemical analyses of apoptosis-associated molecules Bcl-2, Bax, and cleaved-caspase-3 in mouse ovarian tissues with quantification. For statistical significance in this figure, *∗P* < 0.05 compared with young control group; ^#^*P* < 0.05 compared with aging model group; Kruskal-Wallis H test.

Real-time PCR analysis showed that the mRNA expression of antiapoptotic protein Bcl-2 was decreased, but the mRNA expression of proapoptotic protein Bax was increased in the ovary of aging mice; however, PFC significantly restored Bcl-2 mRNA expression but diminished Bax mRNA expression (Figure 3(a)). Western blot analyses confirmed the changes of Bcl-2 and Bax expression at the protein levels in the ovary of the three groups of mice (Figure 3(b)). Additionally, the protein abundance of cleaved-caspase-3 was significantly increased in the ovary of aging mice, but was abrogated by PFC (Figure 3(c)). Immunohistochemical analyses with ovarian tissues gave consistent results on the changes of the above apoptosis regulatory molecules (Figure 4). Altogether, these findings suggested that PFC prevented apoptosis of ovarian GCs in aging mice.

## 4. Discussion

Understanding menopause-associated pathologies and developing novel approaches to manage perimenopause symptoms are important topics in current clinical context. The decrease in estrogen secretion is closely related to the occurrence of perimenopause symptoms. Thus, a commonly used model of perimenopause symptoms can be established via ovariectomy, but this model makes estrogen undetectable [17]. Actually, there is a gradual change of hormones in natural menopause that begins to appear at perimenopause and alters over this transition prior to reaching postmenopause. Ovariectomy also eliminates some other hormones that are likely to play critical roles in menopause; especially, the central modulators of LH, FSH, and gonadotropin-releasing hormone are depleted [18]. Given that the rodents, similar to human, undergo natural hormonal fluctuations in the middle age [19], we used a naturally aging mouse model for experiments in current study, because this model allows for the retention of ovary with an apparent transitional period and thus can more accurately replicate the physical process of aging-associated decline of female reproductive system in human. To eliminate the possible bias introduced in the results, we carefully checked the estrus cycle of each mouse before and during the experiments, because the stages of estrus cycle analogous to menstrual cycle in humans determine the type and sizes of follicles and hormonal levels. We used the mice with regular estrus cycle for experiments and made sure that the mice in each group were at similar stages of the cycle when they were euthanized for examinations.

Over the past decades, investigations of menopause and HRT have produced a great deal of confusion about their effects on women's health. For instance, the evidence for HRT's neuroprotective effects for cognitive disorders in menopausal women was mixed [20]. However, HRT initiated during perimenopause reduced the risk of dementia and had cognitive benefits [21]. Natural products have increasingly been valued as therapeutic opportunities for human diseases. Many studies have reported that PFC had antiaging effects. For example, some studies examined the effects of PFC on D-galactose-induced aging in rats and found that PFC at 0.28 g/kg by gavage for 30 days increased the activity of superoxide dismutase, inhibited lipid peroxidation, and upregulated the mRNA expression of nerve growth factor mRNA [9]. It was also reported that treatment with PFC-containing rabbit serum at a low concentration of 1.5% for 48 h exerted antiaging effects on human dermal fibroblasts cells* in vitro* [9]. All these findings promoted us to ask whether PFC could improve ovarian function in aging-associated perimenopause symptoms. Indeed, we observed that PFC improved ovarian histology and hormone secretion in aging mice. Notably, PFC downregulated the serum concentrations of LH and FSH, two important hormones secreted by gonadotropic cells of the anterior pituitary gland [22], which were consistent with the common phenomenon of high serum levels of LH and FSH in women after menopause because of the decreased feedback control of pituitary secretion due to the low levels of estradiol and progesterone. Additionally, we observed that the expression of IGF-1 was reduced in the ovary of aging mice but upregulated by PFC treatment. IGF-1 has been implicated in follicle development and is considered to mediate the actions of gonadotrophins and growth hormone at the ovarian level [23]. IGF-1 can be secreted by GCs irrespective of their progestogenic status. In aggregate, our data revealed that PFC might exert systemic regulatory effects probably including the effects on central hormone secretion in aging mice and that regulation of growth factor system was also involved in PFC improvement of ovarian function.

We further performed molecular examinations to elucidate the mechanisms underlying PFC's effects. Our data suggested that apoptosis of GCs was possibly involved in the decreased ovarian function in aging mice. Specifically, Bcl-2 expression was decreased and Bax expression was increased, suggesting the activation of mitochondrial apoptosis pathway. Indeed, mitochondrial dysfunction has been implicated in cellular senescence in general and, in particular, ovarian aging [24]. Recent studies have validated this association by studying mitochondrial DNA copy number as a potential biomarker of embryo viability and the use of mitochondrial nutrients and autologous mitochondrial transfer as a potential treatment for poor ovarian function and response [24]. Although apoptosis occurs as a physiological process in the ovary, being highly important throughout the phases of follicular development, apoptosis in dominant follicles may interfere in ovulation and oocyte quality [25]. Therefore, excessive apoptosis of GCs definitely has negative effects on ovarian function during perimenopause symptoms [8]. Our current findings indicated that PFC considerably reduced apoptosis of GCs in the ovary of aging mice, which could be the molecular basis for PFC improvement of ovarian histology and function. Further experiments are required to determine whether other apoptotic mechanisms were involved in PFC's effects.

## 5. Conclusions

Our data demonstrated that PFC effectively improved the ovarian function in aging-associated perimenopause symptoms through improving histology and hormone endocrine, which could be associated with inhibition of apoptosis of GCs. We suggested PFC as a promising therapeutic option for perimenopause symptoms.

## Data Availability

The data used to support the findings of this study are available from the corresponding author upon request.

## Abbreviations

FSH: Follicle stimulating hormone
GAPDH: Glyceraldehyde phosphate dehydrogenase
GC: Granulosa cells
HE: Hematoxylin-eosin
HRT: Hormone replacement therapy
IGF-1: Insulin-like growth factor 1
LH: Luteinizing hormone
PFC: Polysaccharides of* Fructus corni*.
